# A Médecins Sans Frontières Ethics Framework for Humanitarian Innovation

**DOI:** 10.1371/journal.pmed.1002111

**Published:** 2016-09-06

**Authors:** Julian Sheather, Kiran Jobanputra, Doris Schopper, John Pringle, Sarah Venis, Sidney Wong, Robin Vincent-Smith

**Affiliations:** 1 Ethics Department, British Medical Association, London, United Kingdom; 2 Manson Unit, Médecins Sans Frontières, London, United Kingdom; 3 Medical Faculty, University of Geneva, Geneva, Switzerland; 4 Centre for Education and Research in Humanitarian Action (CERAH), Geneva, Switzerland; 5 McGill University, Montreal, Canada; 6 Médecins Sans Frontières, Amsterdam, The Netherlands; 7 Médecins Sans Frontières, Brussels, Belgium

## Abstract

Kiran Jobanputra and colleagues describe an ethics framework to support the ethics oversight of innovation projects in medical humanitarian contexts.

**Summary Points**

- Humanitarian organisations often have to innovate to deliver health care and aid to populations in complex and volatile contexts.
- Innovation projects can involve ethical risks and have consequences for populations even if human participants are not directly involved. While high-level principles have been developed for humanitarian innovation, there is a lack of guidance for how these should be applied in practice.
- Médecins sans Frontières (MSF) has well-established research ethics frameworks, but application of such frameworks to innovation projects could stifle innovation by introducing regulation disproportionate to the risks involved. In addition, the dynamic processes of innovation do not fit within conventional ethics frameworks.
- MSF developed and is piloting an ethics framework for humanitarian innovation that is intended for self-guided use by innovators or project owners to enable them to identify and weigh the harms and benefits of such work and be attentive towards a plurality of ethical considerations.

## Background

**Fig 1:**
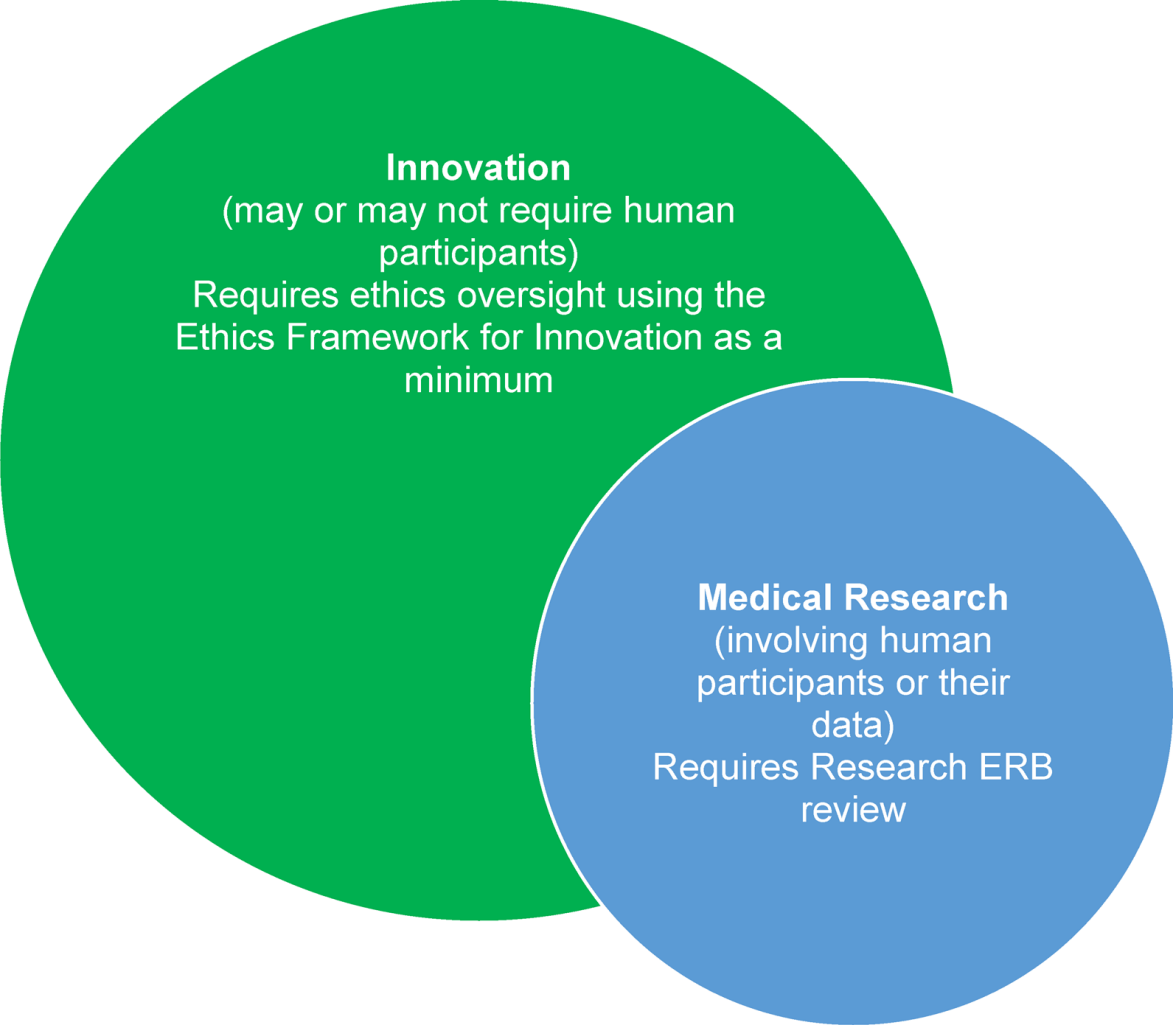
Relation between humanitarian innovation and medical research and their oversight in MSF.

Innovation is at the core of humanitarian action. Humanitarian contexts are often volatile, uncertain, complex, and ambiguous, requiring responders to take a flexible, learning approach. For a medical humanitarian organisation such as Médecins sans Frontières/Doctors Without Borders (MSF), the challenges of delivering assistance to people in need mean that innovation is an ethical obligation; we cannot rely on old solutions for new problems. For instance, the unprecedented scale of the West African Ebola outbreak presented challenges in patient and data management that required rapid adoption of new tools and methods of working [1,2]. For MSF, innovation involves the creation or implementation of new products or processes to improve quality of care and become more effective in pursuit of our goals of providing medical assistance and “témoignage” (witnessing) to populations in need [3]. Along with intended benefits, however, can come harms, either to the individuals or populations we seek to benefit; to our staff, processes, and reputation; or to the trust placed in us by those in need (Box 1). Well-established ethical guidance exists for medical research in humanitarian settings [4–10]. However, many innovations fall outside the purview of formal research ethics review, and there is a need for ethics guidance for innovations specific to humanitarian action. To address this issue in MSF, we have developed and piloted an ethics framework for innovation projects. Fig 1 outlines the relationship between innovation and research in MSF and clarifies when use of the framework would be appropriate. Research involving human participants, including use of their data, requires approval by the MSF Ethics Review Board (ERB) [6,7], and, similarly, innovation projects that constitute medical research (i.e., involving human participants or their data) should follow the research ethics review process.

**Box 1. Harms That May Arise from Innovation Projects**

- Privacy harms: these include inappropriate use, transfer, or storage of personal data.
- Failure to consider the impact of the innovation on the culture, attitudes, or values of the target populations (which may be heterogeneous).
- A failure to engage appropriately those likely to be affected by the innovation. Humanitarian innovation must be rooted in a respect for dignity. Parachuting innovations into complex environments without working collaboratively with affected individuals and populations can be perceived as patronizing, undermine trust, and result in failure. It can also lead to wrongs if innovations produce commercial benefits that are not shared with the community.
- A failure to consider local solutions. Humanitarian interventions are often characterized by a significant power differential. A failure to identify and (where appropriate) build on local solutions could jeopardize the acceptance and sustainability of an innovation.
- Harms to the organization responsible for the innovation, which may subsequently compromise the ability to deliver aid. These include:
  - Threats to trust: interventions cannot succeed without the trust of the recipient individuals and populations.
  - Reputational harms (closely linked to trust). Humanitarian agencies depend on their reputations as trusted independent, neutral, and impartial providers of medical relief. Innovation can involve working with nontraditional partners, including commercial providers, governments, or militaries. As such, reputational risks can arise as a result of conflicting partnerships, when partners have agendas that may be—or may be perceived to be—antagonistic to humanitarian goals. Likewise, conflicts of interest may occur when innovators have personal (nonhumanitarian) interests in the innovation.

The framework is intended for self-guided use by nonmedical innovators (or innovation project owners) with little or no knowledge of medical ethics. Our hope is that it will provide a useful tool to organisations undertaking innovative projects in humanitarian settings by enabling staff to identify and weigh the harms and benefits of such work and be attentive towards a plurality of ethical considerations.

### The Need for a Framework

Why not just use existing research ERBs for innovation projects? Importantly, many humanitarian innovation projects do not directly involve human participants or their data, even if they may ultimately have an impact on patient care or services provided for communities. For such projects, the approach to ethics oversight should be proportionate to the reasonably foreseeable harms. In seeking innovative solutions, ethical judgements may need to be made that require potential benefits and harms to be identified and weighed. However, excessive or disproportionate regulation could stifle innovation such that potential benefits could be lost [11]. MSF, as an organization, wants to create an environment conducive to innovation without imposing excessive or disproportionate bureaucratic oversight. This tension between the imperative to innovate and the need to avoid or mitigate harm was key to the decision to develop our ethics framework. Furthermore, innovation is a dynamic process, seldom a one-off event. It involves identifying problems, developing and selecting possible solutions, preliminary implementation and testing, and, where merited, widespread adoption [12]. An ethics framework for innovation should therefore not be an initial hurdle to be jumped but a guide that promotes and informs reflection throughout the innovation cycle.

### Deriving an Ethics Framework for Humanitarian Innovation

The framework was developed through an iterative discursive process between senior MSF medical and operations managers and bioethicists with experience in humanitarian medical programming and service delivery. The starting point was our core humanitarian values of neutrality and impartiality [3]. The emerging framework was reviewed in light of literature on biomedical and research ethics as well as ethics of humanitarian innovation [3,4,11,13–16]. Two resources were particularly important: firstly, the MSF Research Ethics Framework, which elaborates key concerns in planning and enacting research, some of which are translatable to innovation projects [4]; secondly, recent elaborations of high-level ethics principles in humanitarian innovation [14,15]. However, one issue that arises when using exclusively high-level principles is the absence of decision-guiding content in specific circumstances—they can leave moral judgment “undetermined” [16]. A major challenge with a principled approach is therefore stepping down from high-level principles to practical decision-making. We thus decided that our framework should capture the moral concerns of the innovation principles from an MSF vantage point that includes the foundation of the MSF Research Ethics Framework [4]. Our intention is to facilitate and focus MSF innovation and thus to be practically oriented in guiding action.

We give particular emphasis to promoting a participatory approach to innovation, as the relationship between humanitarian organisations and the populations they assist can be the source of ethical tensions and opportunities. This also informs the focus of the framework on the needs of particularly vulnerable groups—a central moral concern for MSF. Our framework is not intended to give simple binary outcomes. This is where the hard work of ethical judgment takes place. It involves specifying which of the principles are active in any given decision—that is to say, what the respect for any individual principle means in the context under discussion—and the strength of the claims arising from it. It may also involve weighing the claims arising from different principles and adjudicating between them where they conflict.

### A Médecins Sans Frontières Ethics Framework for Humanitarian Innovation

This framework is intended to be used to guide work that does not directly involve human participants and does not lie within the purview of formal research ethics oversight.

1. Clearly **identify the problem** you are seeking to address and what benefit you expect the innovation to have. This step may seem obvious, so what is its ethical significance? When identifying the problem, there should be consideration of upstream solutions that may address the problem in a holistic and sustainable way. For instance, rather than focusing on technocratic fixes, what are the sociopolitical determinants of the problem and the wider possibilities for solutions? Who has stakes in finding a solution and who may have interests in perpetuating the problem? Is the problem a moving target? Collaboration and cross-fertilization with other disciplines should be considered in order to help to see the problem from various perspectives. In short, do not underestimate the importance of fully identifying the problem.
2. **Ensure that the innovation shows respect for human dignity.** While this is a broad concept, it has practical implications. The focus of concern is respect for human beings, reminding us that the simplest or most direct solutions may not be ethically appropriate. Innovators must show due respect for the multiple and overlapping interests of those affected by the innovation. It extends beyond a concern for physical wellbeing to include psychological and cultural integrity. It also incorporates a concern for individual privacy and a respect for the confidentiality of individual-, family-, and community-based data.
3. **Clarify how you will involve the end user** from the start of the process. Innovation should be driven by the requirements of the user. The innovation cycle should be participatory, using methods to involve relevant individuals and communities. Innovators must be sensitive to power dynamics between and within cultures and power imbalances between aid workers and beneficiaries.
4. **Identify and weigh harms and benefits.** When considering innovations, a critical first step is the identification, as far as is reasonably possible, of potential harms along with the anticipated benefits. The next step involves weighing these harms and benefits.
  - Where reasonably foreseeable harms outweigh the likely benefits, implementation will not be ethical. Potential harms include, but are not limited to, physical and psychological harms to individuals. There is also need to consider potential harm to communities.
  - Where innovation involves a favourable balance of benefits and harms, all reasonable steps must be taken to minimise (mitigate) the harms as far as possible. Unnecessary harms must be eliminated. Where harms are unavoidable, those affected should be informed of the nature and severity of the risks involved.
  - Conflicted partnerships or conflicts of interest may result in reputational harm to the organisation. If these are identified, then oversight by an existing ERB is recommended.
5. **Describe the distribution of harms and benefits, and ensure that the risk of harm is not borne by those who do not stand to benefit.** Innovators need to give careful consideration to the distribution of benefits and harms associated with their projects. Do the risks or benefits fall unequally across groups? If so, is it appropriate to proceed, and how can these inequalities of distribution be addressed or mitigated? Equally, it is important that the innovation takes into account vulnerable groups; it may be ethically warranted to give particular attention to those who have particular needs. Just as we tend to give more health care to the unwell, so particular attention may need to be given to those who are vulnerable or who may not be able to protect their own interests. This is expressed in the humanitarian principle of impartiality. In addition, consider whether anyone is “wronged” by the innovation. A “wrong” is an infringement that is distinct from harm. For example, selecting one group for an innovation project over another may wrong the other group (as opposed to harming them).
6. **Plan (and carry out) an evaluation that delivers the information needed for subsequent decisions to implement or scale-up the innovation, and then ensure that the beneficiaries have access to the innovation.** Innovation requires an acceptance of the risk of failure—not all innovation projects will achieve their desired outcome. But in all cases, we can learn and apply these lessons in the future. Given the time, energy, and resources that these projects require, rigorous evaluation and sharing of lessons is itself a moral obligation. Therefore, consideration should be given to dissemination of findings, since it may be important to avoid further exposure to potential harm by sharing findings, whether these are positive or negative. Likewise, there should be a willingness and strategy for wider implementation of the innovation if found to be successful and a commitment to ensure beneficiaries—at least in the communities where it was tested and ideally in similar communities affected by humanitarian crises—have access to the innovation subsequently.

### Applying the Framework

The case studies presented in Tables 1–3 are based on analysis of abstracts and slides of conference presentations of MSF innovation projects [17–19]. Project leaders were contacted and gave permission for their project to be analysed and to provide clarification where necessary. Each case study contains a brief outline of the project, analysis of the project using the innovation ethics framework, and conclusions about the ethical considerations raised and what might have been done differently if the framework had been applied at the start of the project. Application of the framework identified ethical concerns in all three of the innovation projects. In addition, the conclusion is reached that many of these concerns could have been mitigated had the framework been applied initially. From discussions at fora such as the annual Humanitarian Innovation Conference [20], it is clear that these issues are not unique to MSF but are common across the humanitarian sector.

**Table 1:** New technology for an old disease: unmanned aerial vehicles for tuberculosis sample transport in Papua New Guinea [15].

**Unmanned aerial vehicles (UAVs) for tuberculosis sample transport in Gulf Province, Papua New Guinea**
The transport of diagnostic sputum samples in Gulf Province, Papua New Guinea (PNG), is extremely challenging because of lack of road access. With the agreement of the PNG authorities, the use of unmanned aerial vehicles to transport such samples was trialled in 2014. Although no systematic data collection was conducted, several successful pilot flights were carried out, delivering samples from a remote health facility to the laboratory in Port Moresby. However, the distance of flight was limited to 28 km because of short battery life.
**1. Identify the problem**
The problem that this innovation project addresses is clearly stated, and expected benefits of UAVs in this context is identified.
**2. Respecting human dignity**
•How respectful of individuals and the community is the intervention? The local community was widely informed about the activity and was supportive. The involvement of the local community is a must in the use of drones for civil use in any area. • If health data are being transported along with the samples, has thought been given to possible confidentiality issues? In this pilot, no real samples were transported. The question of how much risk is allowable will be important if this approach is adapted operationally.
**3. Involving the end user**
• Have relevant communities been involved in decisions regarding deployment, timetabling, or flight lines as appropriate? Coordination with the Civil Aviation Safety Authority, local authorities, and all required permissions were obtained. All levels of authorities were supportive. • Have the legal and regulatory issues in relation to the use of UAVs in the proposed area been properly addressed? Flights were conducted through nonpopulated areas at low altitude. All authorities approved the flying schedules. No specific regulation for UAVs was in existence in the country.
**4. Identifying and balancing benefits and harms**
• A successful trial would provide the possibility for rapid scaling and wider implementation. • The potential benefits include rapid collection and testing of samples, which benefits both infected individuals and affected populations and results in increases in efficiency. • Has thought been given to what would happen if the UAVs were carrying highly infectious material and crashed or were downed? An outer, crash-proof case around the samples was used; no infectious material was carried in this pilot. Further evaluation would be needed before hazardous material was transported. • In conflict zones, would they be associated with military UAVs and generate suspicion and resistance? Could this entail risks for staff or reputational risks for MSF? In this setting, there is no history of military use of drones. • Are the UAVs purchased from military suppliers and will this involve reputational risks? In this case, the supplier is civilian with no military connections. • Have the risks associated with mechanical failure been addressed? The UAV used was small and flying over unpopulated areas. For operational use, issues around transport of hazardous material would need to be addressed, as noted.
**5. Consider the distribution of harms and benefits**
• How would different communities respond to the use of these UAVs? The community here has no negative experience of drones and the authorities are supportive. • Would the benefits and harms fall on the same populations or be distributed differently? • Could people, including vulnerable groups such as children, gain access to the material and be infected or otherwise harmed? (For instance, if UAVs crashed because of power or mechanical failure and were retrieved by children.) This risk would need to be assessed before transport of hazardous material.
**6. Evaluation and subsequent implementation plan**
• No evaluation or scale-up was mentioned in the abstract. This was an early-stage pilot and probably did not aim to address all questions relevant to subsequent implementation.
**Conclusion**: insufficient information was provided to assess whether the relevant ethical issues have been identified and managed appropriately—the authors should address this as a significant shortcoming.
**If the framework had been applied from the start of the project**: more effort may have been made to address issues of confidentiality and risk mitigation; means of evaluating the innovation may have been identified.

**Table 2:** The Niger REFRESH borehole project: a paradigm change [16].

***Refresh* project—regenerating damaged or contaminated water boreholes**
Sustainable access to potable water is a vital aspect of many of our programmes. Frequently, this can be achieved only by drilling boreholes down into the aquifer, often at considerable depth. Boreholes are expensive to drill. Unless properly maintained, they can also degrade. They are liable to chemical and biological contamination, physical blockage, and fracture of the casings. They can also be breached by plant and tree roots. The solution has traditionally been to drill a new borehole. This is expensive. Trialling is underway of a cheaper option involving the identification of poorly-performing boreholes, investigation and diagnosis of the problems, and (where appropriate) regeneration of boreholes. This can involve removing blockages by air-lift pumping and addressing water quality by chemical treatment, scrubbing, and flushing.
**1. Identify the problem**
• The problem was clearly identified, and the expected benefits of the intervention were described.
**2. Respecting human dignity**
• Not applicable.
**3. Involving the end user**
• The requirements of the end user are integral to the innovation, but there are questions about the extent to which the technology and expertise can be rapidly and effectively transferred to the local population.
**4. Identifying benefits and harms**
• Financial savings of regeneration make a strong initial case for the project. • The innovation does not directly expose end users to harm—the question is whether the water is safe to drink; this can be scientifically established before use.
**5. Consider the distribution of harms and benefits**
• Not applicable.
**6. Evaluation and subsequent implementation plan**
• Some cost analysis was carried out, showing that this approach offered significant cost savings relative to digging new wells. • It is not clear that this project addressed all questions necessary to decide on implementation.
**Conclusion:** this is an example of an innovative approach to a specific problem that raises no significant ethical concerns. Human participants are not directly involved—or obviously at risk—and the potential benefits significantly outweigh the harms.
**If the framework had been applied from the start of the project**: more thought may have been given as to how this technology and expertise could be transferred to the local community and what evaluation information would be needed to enable decisions on wider implementation.

**Table 3:** Mobilisation of local people and technology in mapping for the Sierra Leone Ebola epidemic response [17].

**Mobilisation of local people and technology in mapping for the Sierra Leone Ebola epidemic response**
During the Ebola epidemic in Sierra Leone, MSF encountered difficulties in rapidly locating villages in which cases of Ebola infection and contact had been identified. There were villages with similar names in different chiefdoms and villages with alternate names. New villages, and some satellite villages, were missing from maps completely. In Tonkolili District, Sierra Leone, MSF trialled an innovative method of gaining accurate information about the location and identity of villages and the availability of local health facilities. Using local “okada” motorbike drivers and local people with GPS-enabled mobile phones, information was gathered across the district about the name, GPS location, chiefdom, ward, and constituency of individual villages. Alternate names, the name and contact number of the village chief or head, and the number of houses in the village were also recorded. Information about the nature and location of any available local health services and the contact details of the local health care workers were also recorded. This information was processed using open-source mapping software to develop accurate and up-to-date maps of the district.
**1. Identify the problem**
The problem was clearly identified, and the expected benefits of the intervention were described.
**2. Respecting human dignity**
• Enrollment of local populations not only in gathering information but also agreeing to its collection and use is important. • Among the potential questions the project raises are the security of the data and the consent of any individuals whose identifying data are captured. • Where identifiable information is being recorded or transferred, appropriate methods for seeking consent need to be explored.
**3. Involving the end user**
• One of the great strengths of the project was its ability to use locally appropriate technology in genuine partnership with local people.
**4. Identifying benefits and harms**
• Volunteers were asked to travel to areas affected by the Ebola epidemic, thus increasing their exposure. This potential harm was mitigated by daily health education briefings that included information on the following: no-touch policy, no contact with objects or surfaces that might have been in contact with sick people, self-assessment (report any headaches or other symptoms), drinking adequate MSF-supplied bottled water, hand washing, etc. • Increased risk of infection to the community. Mitigated by the approach described above and weighed as similar risk to that of outreach workers. • The teams were at risk of being exposed to violence as a result of fear of travellers, particularly those associated with Ebola treatment. This potential harm was mitigated by mappers being instructed to politely ask permission of all village authorities to conduct their surveys and to never argue if asked to stay away or to leave. • Reputational risk to MSF or backlash against the Ebola management centre (EMC) if the behaviour of mappers was not in line with MSF policy. Mitigated by the approach described above. • The benefits to the overall Ebola epidemic response of accurate mapping were significant. The financial cost of the project was modest in relation to the utility of the information. • If this technology were used in conflict zones, for example, it might create anxiety about the data falling into the wrong hands. This potential harm can be to an extent managed by the use of secure MSF servers.
**5. Consider the distribution of harms and benefits**
• There might be some concern that harms were concentrated on the recruited drivers.
**6. Evaluation and subsequent implementation plan**
• No formal evaluation but good analysis of lessons learnt and quality of data collected. • It is not clear what the ultimate implementation plan was (in case of a successful pilot).
**Conclusion:** this innovation project raises some ethical concerns in relation to confidentiality, consent, and data security, which would need to be addressed. However, it should be acknowledged that this is not clinical data and is thus less sensitive an issue. Ultimately, the likely benefits of this innovation significantly outweigh potential harms.
**If the framework had been applied from the start of the project**: more effort may have been made to address issues of confidentiality, consent, and data security and more attention paid to evaluating the innovation to decide on wider implementation possibilities.

## What Next?

The framework has been shared widely within MSF and made available on the submission site for the MSF Scientific Days conference in May 2016 [21]. All authors submitting an abstract on an innovation project were required to confirm either that the project had undergone formal ethics review (in the case of projects involving formal research with human participants or their data) or that the author had used the framework to critically appraise and oversee their own project together with the project sponsor. The framework as presented here represents a first attempt to generate a robust but user-friendly tool to support the ethics oversight of these innovation projects. We intend to evaluate its use and assess its utility through auditing innovation abstracts and surveying their authors as well as through sharing and conversing with other innovators within and outside MSF.

We believe that this framework will help to improve the quality and learning potential of innovation projects as well as the anticipation of potential harms and (where appropriate) their mitigation. Far from discouraging innovation, we hope that this framework will contribute towards a culture of responsible and informed medical humanitarian innovation.

## Abbreviations

EMC: Ebola management centre
ERB: ethics review board
MSF: Médecins sans Frontières
PNG: Papua New Guinea
UAV: unmanned aerial vehicle
